# Bioinspired Reinforcement of Magnetic Colloidal Gel for the Embolization of Hepatocellular Carcinoma

**DOI:** 10.34133/research.1446

**Published:** 2026-10-23

**Authors:** Chaowei Lu, Guangyi Luo, Huai Yang, Jinlong Hu, Xu Yan, Liang Dong, Baoqiang Cao, Yang Lu

**Affiliations:** ^1^ School of Chemistry and Chemical Engineering, Hefei University of Technology, Hefei, Anhui 230009, P. R. China.; ^2^ Anhui No.2 Provincial People’s Hospital, Hefei 230041, P. R. China.; ^3^ Zhejiang Cancer Hospital, Hangzhou Institute of Medicine (HIM), Chinese Academy of Sciences, Hangzhou 310022, P. R. China.

## Abstract

Transarterial embolization is a vital treatment for hepatocellular carcinoma. However, clinically used embolic agents are prone to rapid recanalization and nonspecific embolization. Although colloidal gels composed of nanoparticles are acknowledged for their considerable potential as vascular embolic agent, these gels encounter challenges of long-term and stable embolization in arteries with high blood flow velocities due to their weak mechanical properties. To address the difficulties, the in situ reinforcement strategy was developed to enhance the embolic pressure of colloidal gels. Inspired by plant roots secreting organic acids to dissolve calcium carbonate and absorbing calcium ions, ultimately strengthening the cell wall, alginate and calcium carbonate were incorporated into the colloidal gel composed of gelatin and iron oxide nanoparticles to create a bioinspired reinforced hydrogel. Calcium carbonate nanoparticles dissociated to release Ca^2+^, which cross-linked with alginate, thereby enhancing the mechanical properties of the gel. The bioinspired reinforced hydrogel exhibited smooth extrudability and stable embolization. Additionally, the introduction of iron oxide endowed the hydrogel with magnetic field responsiveness, enabling tumor hyperthermia. Given the unique advantages, the bioinspired reinforced hydrogel holds a promise as novel embolic agents for hepatocellular carcinoma treatment.

## Introduction

Transarterial embolization (TAE) serves as a critical treatment for hepatocellular carcinoma (HCC), selectively occluding tumor-supplying blood vessels [1–4]. This procedure deprives cancer cells of oxygen and nutrients by occluding hepatic artery branches, while normal liver cells remain unaffected due to continued blood supply from the portal vein [5,6]. Commercial embolic agents are broadly classified as solid embolic agents (e.g., polyvinyl alcohol microspheres and gelatin sponge particles) and liquid embolic agents (e.g., lipiodol) [6,7]. However, solid embolic agents are associated with several inherent limitations. These include insufficient homogeneous filling of irregular tumor vasculature and a propensity for particle aggregation, leading to catheter obstruction and the undesirable embolization of larger, proximal vessels [8–10]. Conversely, liquid embolic agents are more suitable for complex target sites. For example, lipiodol’s excellent flow properties enable rapid delivery into small vessels [11,12]. Nevertheless, its lower embolic strength leads to faster clearance by blood flow, increasing the risk of vascular recanalization within the tumor [13,14].

Compared to commercial embolic agents, injectable hydrogels represent a promising approach that offers enhanced precision in vascular embolization while also serving as drug carriers capable of loading a wide range of anticancer drugs [15–18]. Due to their ability to transform into a low-viscosity liquid state under shear stress, they can be easier to inject into small or complex vascular networks. Upon cessation of the shear force, the material rapidly reverts to its stable gel state [19,20]. Colloidal gels utilize nanoparticles as the building blocks to construct 3-dimensional (3D) networks via a bottom-up self-assembly strategy [21]. Physical colloidal gels are dynamic systems in which the network exhibits a shear-thinning capability under applied stress owing to their noncovalent self-assembly mechanism [22,23]. In our previous work, gelatin nanoparticles (GNPs) self-assembled with other magnetic nanoparticles through electrostatic interactions to form colloidal gels. This system not only demonstrated the necessary shear-thinning property but also introduced a magnetothermal response to an alternating magnetic field (AMF), showing compelling potential for integrated vascular embolization and tumor hyperthermia [24–26]. However, physical colloidal gels are mechanically weak and tend to experience sharp reductions in viscosity at high shear rates [23,27,28]. Consequently, they cannot withstand marked external loads, making them vulnerable to the impact of blood flow or shearing forces after implanting in the vascular, which may lead to gel rupture and ectopic embolization [29,30]. Therefore, it is necessary to develop new enhancement strategies to improve the stability of physical colloidal gels.

In situ method hold significant promise for vascular embolization, as hydrogel precursors can be injected via microcatheters into targeted blood vessels, where they undergo in situ reinforcement [31,32]. Photo-crosslinking and solvent exchange strategies are employed to enhance the mechanical properties of hydrogel in situ for stable embolization [33,34]. Given that the in situ method presents a promising strategy for reinforcing physical colloidal gels, it would be beneficial to enhance their toughness through a safe and efficient trigger mechanism, thereby expanding their potential applications. A notable biological example is the formation of strong calcium–pectate complexes in plant cell walls, where calcium ions interact with pectin molecules to provide robust structural reinforcement (Fig. 1A) [35–37]. This mechanism is critical for enhancing the overall rigidity of plant stems [36,38,39]. This process of cross-linking pectin chains relies on the plant’s ability to absorb calcium ions, particularly in calcareous soil where calcium ions originate from calcium carbonate in the soil [40] . Plant roots (e.g., wheat roots) release organic acids that dissolve calcium carbonate in the soil, releasing calcium ions that are subsequently absorbed by the roots [41–43]. This process increases calcium levels in the plant and further enhances the cross-linking of calcium–pectin complexes [44].

**Fig. 1.**
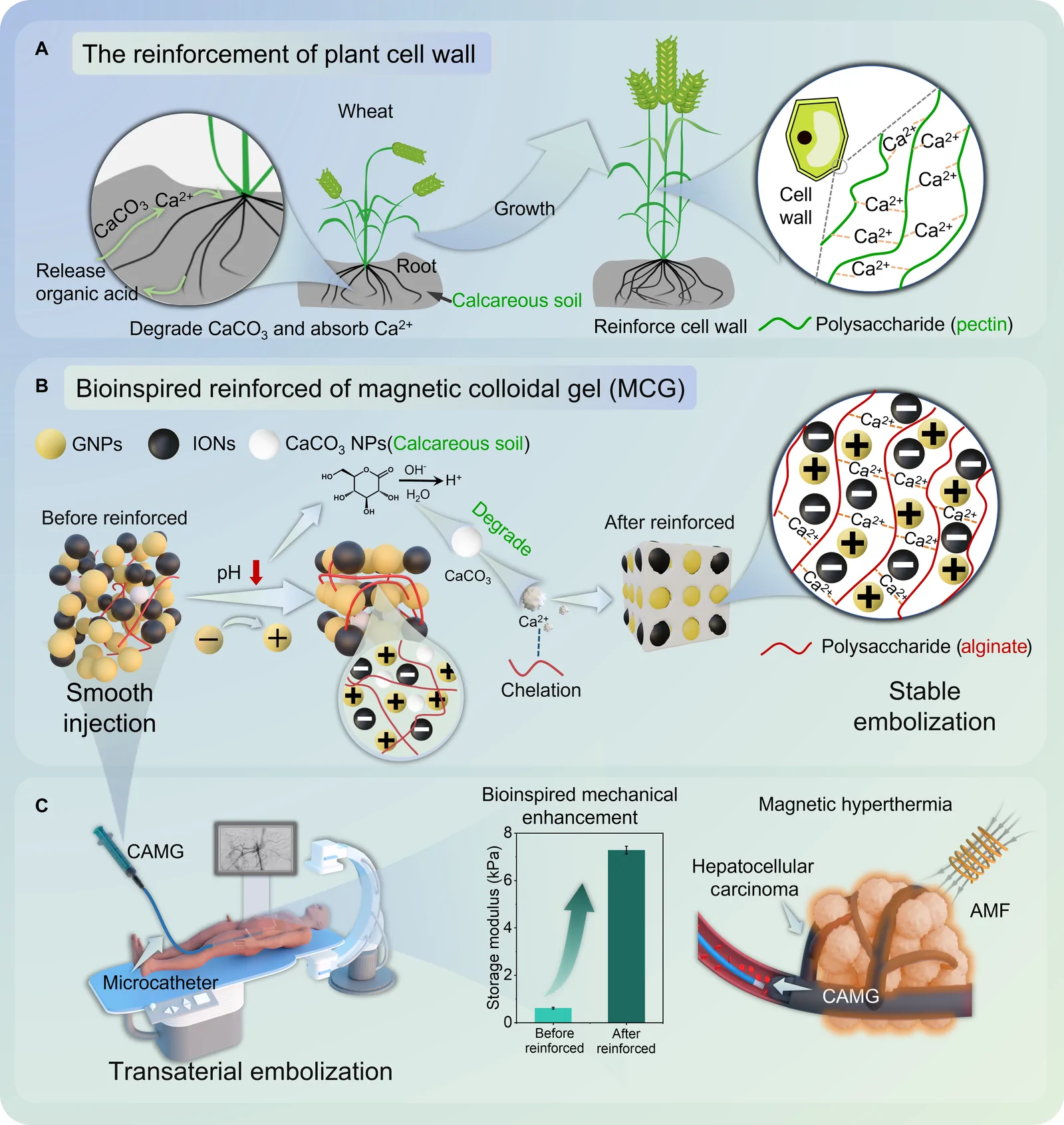
Bioinspired reinforcement strategy of MCG, along with its application in TAE combined with MHT. (A) Wheat roots secrete organic acids to degrade calcium carbonate, absorb calcium ions, and reinforce the cell wall. (B) Preparation of Ca^2+^-chelating alginate (Alg)-enhanced MCG (CAMG). (C) Combination of TAE with MHT for HCC.

Drawing inspiration from the wheat root’s mechanism of organic acid-mediated dissolution of calcium carbonate to enable cell wall strengthening, the present study focuses on the development of a hydrogel with initial smooth extrudability and endovascular reinforced mechanical properties. Sodium alginate (Alg) shares similarities with pectin, particularly in its strong affinity for divalent metal ions [45–47]. This affinity enables Alg to form ion cross-linked networks with divalent ions, a process described by the “egg-box” model, making it ideal for developing biomimetic systems with enhanced mechanical and biological properties [48–50]. In this study, Alg and calcium carbonate nanoparticles (CaCO_3_ NPs) are incorporated into a magnetic colloidal gel (MCG) composed of GNPs and iron oxide nanoparticles (IONs) with the aim of preparing Ca^2+^-chelating Alg-enhanced MCG (CAMG), which is denoted as C*_x_*AMG (*x* = 0.5, 1.0, and 1.5, corresponding to CaCO_3_ NP concentrations of 0.5%, 1.0%, and 1.5% w/v, respectively) (Fig. 1B). All the components of the hydrogel exhibit good biocompatibility, making it a promising candidate for clinical translation. The extrusion through catheter was evaluated to assess the potential of the hydrogel to meet clinical requirements for manual injection. In addition, the storage modulus increased more than 10 times after CAMG reinforcement, along with the embolic effects of the hydrogel in the renal arteries and HCC blood supply arteries. Furthermore, the incorporation of IONs endowed the hydrogel with magnetic field-responsive properties, and the magnetic induction heating induced by AMF showed promise for hyperthermia of HCC (Fig. 1C). The bioinspired reinforced injectable magnetic hydrogel system shows promise as a novel embolic agent for liver cancer treatment.

## Results and Discussion

### Preparation and characterization of hydrogel

The bottom-up self-assembly strategy is commonly used to construct colloidal gel frameworks [51]. As shown in Fig. 2A, GNPs and IONs formed a 3D network through electrostatic assembly. The natural polysaccharide Alg was then introduced to infiltrate and fill the gaps between nanoparticles. Both GNPs and IONs, the building blocks for assembly, were spherical nanoparticles with average hydrodynamic diameters of ~240 nm and ~130 nm, respectively (Figs. S1 and S2). Similarly, the CaCO_3_ NPs used to facilitate the crosslinking of Alg was also rough, spherical particles sized at ~110 nm (Fig. S3). Importantly, the charge-reversal property of the GNPs enables the assembly of colloidal gels, which can be controlled by adjusting the pH of the solution [23–25]. As shown in Fig. 2B, the zeta potentials of the GNPs and IONs were measured under different pH conditions. At pH 11, both the GNPs and IONs initially carried negative charges, enabling them to be uniformly mixed in the aqueous solution. Upon the introduction of d-(+)-gluconic acid δ-lactone (GDL) as an acidifier, the continuous hydrolysis of GDL gradually reduced the pH to ~8, which is below the isoelectric point of the GNPs. Consequently, the surface charge of the GNPs transitioned from negative to positive, promoting electrostatic interactions between the GNPs and the negatively charged IONs to form the MCG based on the preparation illustrated in Fig. 2C. In addition, according to the methods illustrated in Fig. S4, Alg and CaCO_3_ NPs were introduced into the MCG to prepare bioinspired reinforced hydrogel. The scanning electron microscopy (SEM) images of the 3D MCG network structure formed by particle stacking were shown in Fig. 2D (I). In addition, elemental mapping performed following the addition of Alg and CaCO_3_ NPs showed that these species were evenly distributed within the gel matrix (Fig. S5A). Furthermore, as presented in Fig. 2D (II), the microstructure of CAMG exhibited a double network structure, in which Alg chelated with Ca^2+^ to form cross-linking nodes and envelop the particles. Elemental mapping analysis showed that the units were uniformly distributed within the CAMG structure after reinforced (Fig. S5B). To evaluate the strength of the MCG and CAMG, they were molded into cylindrical hydrogels, and their shape changes under external load were compared. As shown in Fig. 2E, the MCG was structurally damaged after applied load and did not recover after removal of the external force. In contrast, CAMG sustained a 20-g weight and exhibited no obvious deformation. To further compare the differences in mechanical properties between the MCG and CAMG, the freshly prepared gels were injected into heart- and pentagram-shaped molds and then allowed to stand for 2 h. After subsequent demolding, CAMG possessed clearer contour details and could be easily picked up with tweezers, in contrast to the MCG (Fig. 2F). These results demonstrated that the double-network structure of CAMG endowed it with superior mechanical properties compared to MCG. As shown in Fig. 2G, the freshly prepared MCG and CAMG exhibited smooth extrudability before reinforced. What is more, the magnetic IONs imparted excellent magnetic responsiveness to the gel. More specifically, when extruded from a syringe in the presence of a magnetic field, the gel was attracted by the magnet, confirming its high sensitivity to magnetic fields (Fig. S6).

**Fig. 2.**
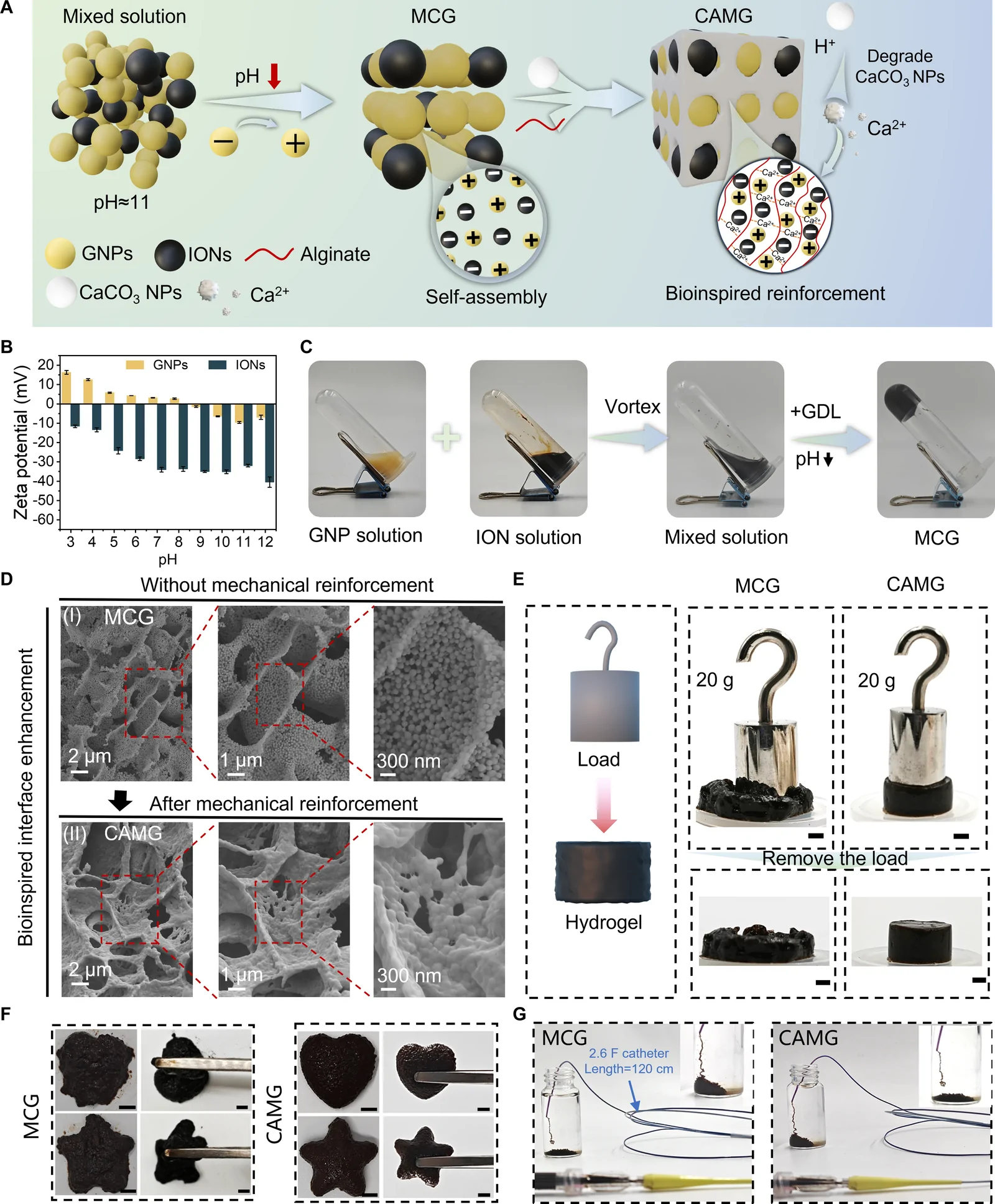
Comparison of the mechanical properties of MCG before enhancement against those of CAMG after enhancement. (A) Schematic representation of the self-assembly strategy for MCG and the bioinspired reinforcement of the colloidal gel. (B) Zeta potential of GNPs and IONs at different pH. (C) MCG was formed through electrostatic interactions. (D) Comparison of SEM images between MCG (prior to mechanical enhancement) and CAMG (after mechanical enhancement). (E) Deformation of cylindrical MCG and CAMG under external force. (F) MCG and CAMG were molded into specific shapes and tested for pick-up with tweezers. (G) Photographs of the freshly prepared MCG and CAMG injected through a 2.6-F catheter. Scale bar, 3 mm.

The facile and smooth injection of embolic agents through a catheter is crucial for clinical procedures. In this context, it was considered that the incorporation of excessive Alg could increase the viscosity dramatically of AMG due to an abundance of 1,4-linked β-d-mannuronic acid units [52,53]. Indeed, using 3% w/v Alg in the prepared AMG, the injection force exceeded 50 N through a 2.6-F catheter (Fig. S7), rendering the manual injection of this gel impossible. Conversely, a lower Alg content of 1% w/v was unable to provide sufficiently strong mechanical strength for resisting the physiological embolic pressure of 16 kPa initially (Fig. S8). Therefore, 2% w/v Alg content was selected for the preparation of the colloidal gel interstitial filler.

As shown in Fig. 2A, CAMG could be injected smoothly through the microcatheter and then reinforced for stable embolization. As shown in Fig. S9, the shear-thinning property of AMG was not affected by the introduction of Alg. Additionally, the viscosity was found to decrease sharply as the shear rate increased for the C_0.5_AMG, C_1.0_AMG, and C_1.5_AMG at 0 min. This suggested a notable shear-thinning property and provided a basis for the injection of these gels through the catheter. From the perspective of clinical applications, it is essential to determine whether an embolic agent can be easily injected manually through a catheter. To assess this, the injection force was tested through 2.6-F (inner diameter = 0.69 mm, length = 125 cm) and 4-F (inner diameter = 0.97 mm, length = 100 cm) catheters using the Material Test System (Fig. S10). The injection forces of the freshly prepared C_0.5_AMG, C_1.0_AMG, and C_1.5_AMG were 12.2 ± 0.1, 12.4 ± 0.3, and 14.8 ± 0.3 N through the 4-F catheter, and 26.8 ± 0.8, 27.8 ± 0.7, and 31.2 ± 0.9 N through the 2.6-F catheter, respectively (Fig. 3B and Figs. S11A and B). These values were all below 50 N, indicating that the gels were suitable for delivery through a catheter [19,24]. Rheological characterization was used to assess whether the hydrogel exhibits enhanced mechanical performance for providing stable embolization. Upon hydrolysis of the GDL to reverse the charge on the GNPs, the mechanical properties of the MCG were enhanced through electrostatic assembly, increasing the storage modulus (*G*′) from 192.2 ± 11.0 to 312.4 ± 27.2 Pa (Fig. 3C). Additionally, using a 2% w/v Alg content, *G*′ of AMG was slightly enhanced to 573.3 ± 15.6 Pa compared to that of the MCG alone, and this was attributed to the increased solid content in the gel system. Upon the introduction of CaCO_3_ NPs to AMG, no significant changes in *G*′ were observed. Notably, *G*′ of C_1.0_AMG was increased from its initial value of 615.7 ± 39.8 to 7,284.5 ± 164.2 Pa after 120 min, representing an over 10-fold increase. This phenomenon was attributed to the acid decomposition of the CaCO_3_ NPs, which generated Ca^2+^ that chelated with Alg, ultimately resulting in a substantial enhancement in the gel mechanical properties. In contrast, at CaCO_3_ NP concentrations of 0.5 and 1.5% w/v, the *G*′ values remained relatively low. It was also found that *G*′ of the C_0.5_AMG increased from 560.2 ± 40.7 to 3,306.4 ± 56.9 Pa, which was attributed to the limited release of Ca^2+^, resulting in a lower degree of cross-linking with Alg and, consequently, a lower modulus. For C_1.5_AMG, *G*′ increased from 709.2 ± 21.3 to 5,376.7 ± 119.7 Pa, which was attributed to the higher CaCO_3_ NP content releasing excessive Ca^2+^, potentially causing nonuniform binding with Alg. This hindered the formation of symmetrical “egg-box” dimers and promoted cross-linking both between and within the alginate molecules, ultimately reducing gel hardness [54,55]. Additionally, frequency sweeps performed at 0 and 120 min (Figs. S12A and B) confirmed the stability of these gels at different frequencies. It was also found that the critical strain decreased from 68.41% to 26.63% as the CaCO_3_ NP content was increased (Fig. S13), indicating a gradual reduction in the ability of the gel to withstand external strain. Based on the above results, C_1.0_AMG showed an injection force of only 27.8 N (below the 50-N manual limit), a >10-fold increase in storage modulus to 7,284 Pa within 120 min, and sufficiently strong mechanical strength for resisting the physiological embolic pressure of 16 kPa initially. In contrast, C_0.5_AMG and C_1.5_AMG suffered from insufficient crosslinking and nonuniform chelation, respectively, failing to combine easy injectability, mechanical reinforcement, and durable embolization. Therefore, C_1.0_AMG was selected as the optimized formulation for its optimal synergy.

**Fig. 3.**
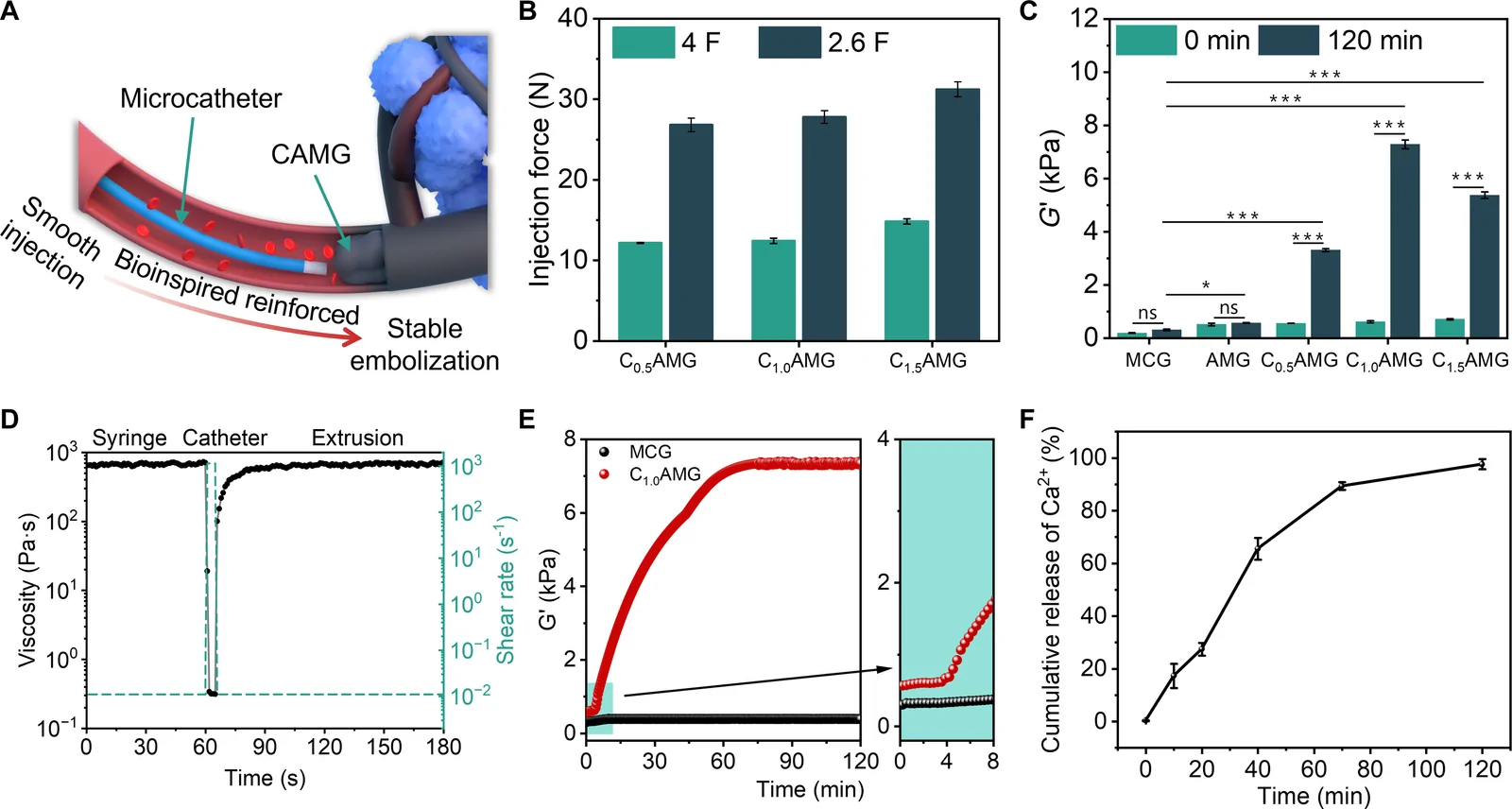
Injection force test and rheological characterization of hydrogel. (A) Diagram of the freshly prepared CAMG exhibiting smooth injectability through the microcatheter and achieving stable embolization after reinforced. (B) Injection force test through 2.6- and 4-F catheters of freshly prepared C_0.5_AMG, C_1.0_AMG, and C_1.5_AMG. (C) Comparison of *G*′ before and after gel reinforced of MCG, AMG, C_0.5_AMG, C_1.0_AMG, and C_1.5_AMG. (D) Peak-hold test simulating flow during extruding through catheter and recovery post-extrusion of freshly prepared C_1.0_AMG. (E) *G*′ versus time for MCG and C_1.0_AMG. (F) Cumulative release of Ca^2+^ over time in CaCO_3_ NPs and GDL mixed solution. *P ≤ 0.05, **P ≤ 0.01, and ***P ≤ 0.001.

To assess viscosity changes during extrusion through a catheter of freshly prepared hydrogel, a peak-hold test was performed. At the start of extrusion, the hydrogel encountered a low shear rate. Subsequently, as the gel passed through the catheter, the shear rate sharply increased, before dramatically decreasing as the gel left the catheter tip. As shown in Fig. 3D, the C_1.0_AMG precursor not only exhibited shear-thinning behavior but also recovered its original viscosity when exposed to the sequential shear rate described above. This behavior provided a foundation for the gradual reinforcement of the mechanical properties of C_1.0_AMG over time and endowed it with the potential to serve as a clinical embolic agent. To further evaluate the enhanced process, a time sweep test was conducted to track changes in the hydrogel’s modulus. As illustrated in Fig. 3E, *G*′ of C_1.0_AMG increased over time and reached a plateau (~7,300 Pa) after ~80 min, suggesting that the mechanical properties of this gel gradually improved over time. Notably, *G*′ did not begin to increase until ~4 min, providing sufficient manipulation time for use in clinical procedures. Beyond this time, the gel within the vessel should progressively harden, leading to effective embolization. The increase in *G*′ of C_1.0_AMG over time was attributed to the gradual hydrolysis of GDL, which led to a corresponding increase in CaCO_3_ NP degradation, and resulted in a gradual increase in the cross-linking density between Alg and Ca^2+^. It was also observed that the structure of the freeze-dried C_1.0_AMG became denser over time, indicating that more abundant cross-linking was present within the hydrogel (Fig. S14). Subsequently, to verify the modulus change over time, the cumulative release of Ca^2+^ and pH variation was measured. It was found that in a mixed solution of CaCO_3_ NPs and GDL, a reduction in the pH correlated with an increase in the amount of released Ca^2+^ (Fig. 3F and Fig. S15). Powder x-ray diffraction (XRD) analysis indicated that after reinforced, C_0.5_AMG, C_1.0_AMG, and C_1.5_AMG exhibited diffraction peaks corresponding only to Fe_3_O_4_, with no trace of CaCO_3_ being observed, and suggesting that the CaCO_3_ NPs were completely degraded (Fig. S16A to C). The above results therefore confirmed that C_1.0_AMG allowed smooth injection through a catheter prior to its subsequent reinforcement, and indicating its potential for application in blood vessel occlusion.

### In vitro embolization performance

A simulated blood circulation system was used to assess the maximum pressure that the hydrogel could withstand in blood vessels. As shown in Fig. 4A, 200 μl of the hydrogel was injected into a silicone tube. Upon initiating the anticoagulant blood flow at rate of 50 ml min^−1^, a significant increase in pressure was observed within the simulated blood vessel. When the pressure exceeded the hydrogel’s maximum tolerance, the gel structure was disrupted, causing a sharp decline in pressure (Fig. 4B). As illustrated in Fig. 4C, the embolic pressure measured with the MCG (12.1 ± 1.6 kPa) was lower than the blood pressure, leading to an inability to embolize the blood vessels. In contrast, the embolic pressure achieved for C_1.0_AMG at 0 min was 22.2 ± 1.2 kPa, which slightly exceeded the physiological blood pressure, but did not allow long-term vascular embolization at this point due to shear thinning properties. However, the degree of chelation between Ca^2+^ and Alg gradually increased over time, and after 120 min, the embolic pressure of C_1.0_AMG reached 69.9 ± 6.0 kPa, which was sufficient to block blood flow in the artery, achieving a stable embolic effect.

**Fig. 4.**
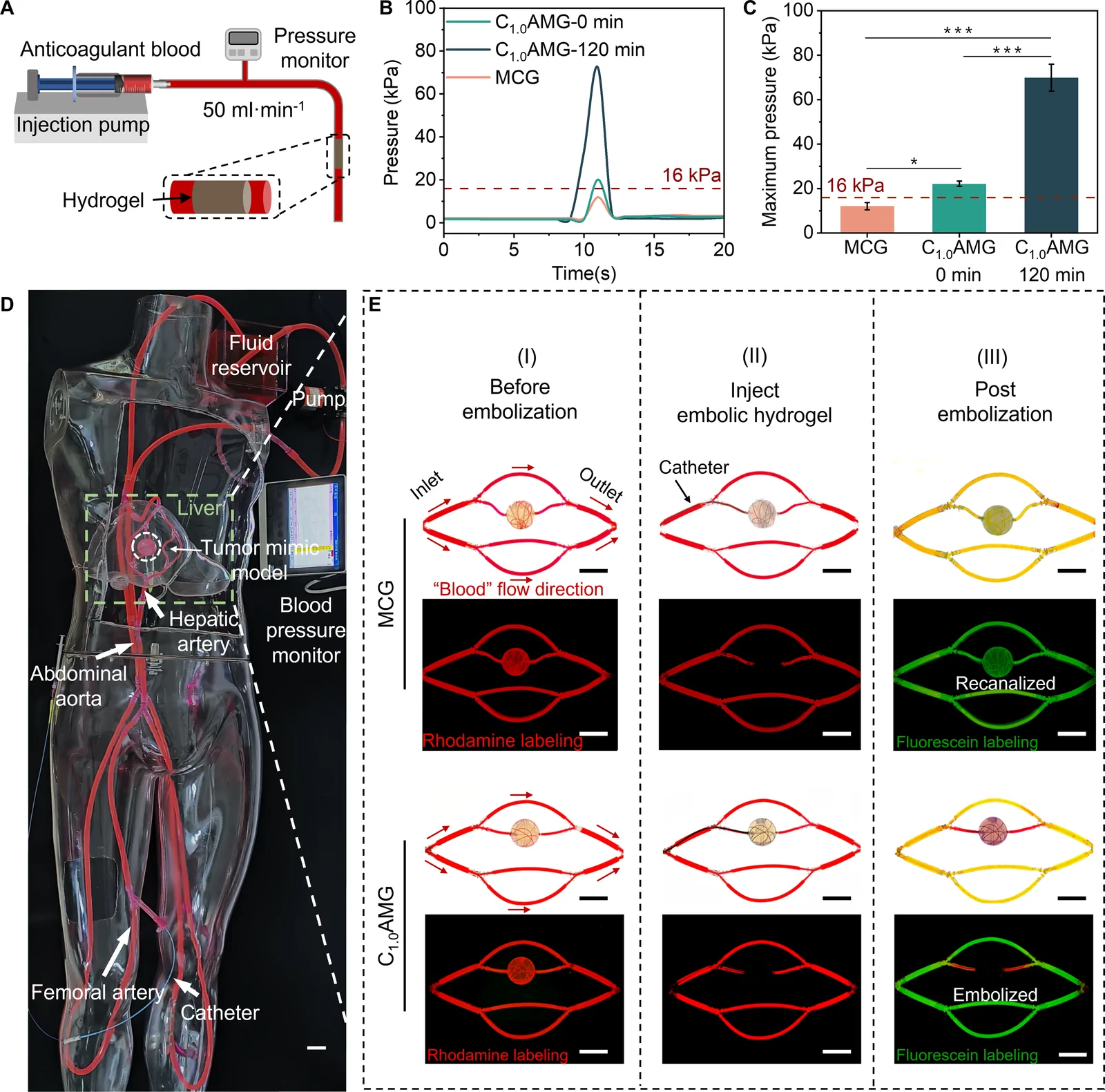
The embolization test of C_1.0_AMG in a simulated blood circulation system. (A) Schematic diagram of embolic pressure test device of the hydrogel. (B) Embolic pressure curve and (C) maximum pressure of MCG and C_1.0_AMG at 0 and 120 min. (D) Digital photo of simulated blood circulation system. (E) Digital photos (upper) and corresponding fluorescent images under 365-nm ultraviolet (lower) of embolization simulation process of hydrogel in the system. The red fluid (represented simulated blood) was rhodamine B, and the corresponding fluorescence showed red; the yellow fluid (represented contrast agent) was fluorescein sodium, and the corresponding fluorescence showed green; the dark substances in the tumor-mimic model were hydrogel. Scale bar, 3 cm. *P ≤ 0.05, **P ≤ 0.01, and ***P ≤ 0.001.

To demonstrate the clinical feasibility of C_1.0_AMG, an in vitro simulated blood circulation system (Fig. 4D) was employed to evaluate the embolization effect of the hydrogel in a tumor-mimic model. The system comprises a mannequin integrated with a blood pump, fluid reservoir, pressure monitor, and 3D vascular system corresponding to the liver tumor-mimic model (including the hepatic artery). An agarose ball filled with silicone tubes (inner diameter = 300 μm) was used to simulate the complex vascular channels in the model. As shown in Fig. 4E (I), red simulated blood (rhodamine B) was introduced into the 3D vascular system prior to embolization. Digital photograph and the corresponding fluorescence image revealed the distribution of the main supplying arteries and the complex network of blood vessels within the tumor-mimic model. Subsequently, freshly prepared MCG and C_1.0_AMG samples were injected into the feeding site of the tumor-mimic model through a microcatheter, as illustrated in Fig. 4E (II). Digital photographic images showed retention of the hydrogels within the complex vascular channels of the tumor-mimic model, causing nonvisualization of the tumor-mimic blood vessels in the fluorescence images. These results confirmed the excellent delivery capabilities of MCG and C_1.0_AMG. After embolization, a yellow contrast agent (fluorescein sodium) was injected into the vessel to assess the effect of embolization after ~1 h [Fig. 4E (III)]. Digital photographic images showed that no obvious hydrogel residue remains inside the tumor-mimic model, while the fluorescence images showed green vascular outlines when MCG was used as the embolic agent. These results indicated that MCG was unable to achieve long-term embolization and led to recanalization of the blood vessels. However, when C_1.0_AMG was used as the embolic material, the digital photographic images and corresponding fluorescence images showed clear retention inside the tumor-mimic model. More specifically, no fluorescence was observed for the internal blood vessels of the tumor-mimic model, and the blood vessels at both ends of the tumor-mimic model remained red, indicating that no liquid could pass through the simulated vessels in the tumor-mimic model, and confirming its embolization effect. These results clearly indicated the potential of C_1.0_AMG for use as a clinical embolic agent.

### In vivo renal artery embolization

The in vivo embolic capability of C_1.0_AMG was subsequently evaluated using a New Zealand rabbit renal artery embolization model (Fig. 5A). Initially, a 4-F microcatheter was superselectively inserted into the abdominal aorta via the femoral artery (FA) approach. Iodixanol (400 μl) was injected through the catheter to observe the vascular trajectory, determine the position of the renal artery, and clearly visualize the significant contrast in both kidneys using digital subtraction angiography (DSA) [Fig. 5B (I)]. Subsequently, the catheter was superselectively inserted into the right kidney artery. Freshly prepared C_1.0_AMG was then injected in combination with iodixanol for intraoperative imaging, which was denoted as C_1.0_AMG-I (Fig. S17A). Rheological characterization and injection force test indicated that iodixanol did not significantly affect the mechanical properties after reinforced or extrudability of the hydrogel (Fig. S17B to D). Notably, during the injection process, the hydrogel flowed along the blood vessels and adapted to their shape. Meanwhile, owing to its initial viscosity, the hydrogel remained lodged in the blood vessels, as confirmed by x-ray imaging, which showed successful filling of the vascular branches and arteries of the kidney [Fig. 5B (III)]. To verify the vascular embolization effect of C_1.0_AMG-I, iodixanol was injected, and the resulting angiography images showed no contrast in the right kidney, confirming the stable embolization effect [Fig. 5B (II)]. On the 28th day post-embolization, computed tomography (CT) was performed to evaluate the volume change of kidneys. (Fig. 5C) Transverse CT and (Fig. 5D) coronal CT images showed significant atrophy of the right kidney, while the left kidney exhibited a compensatory enlargement (Fig. 5C and D).

**Fig. 5.**
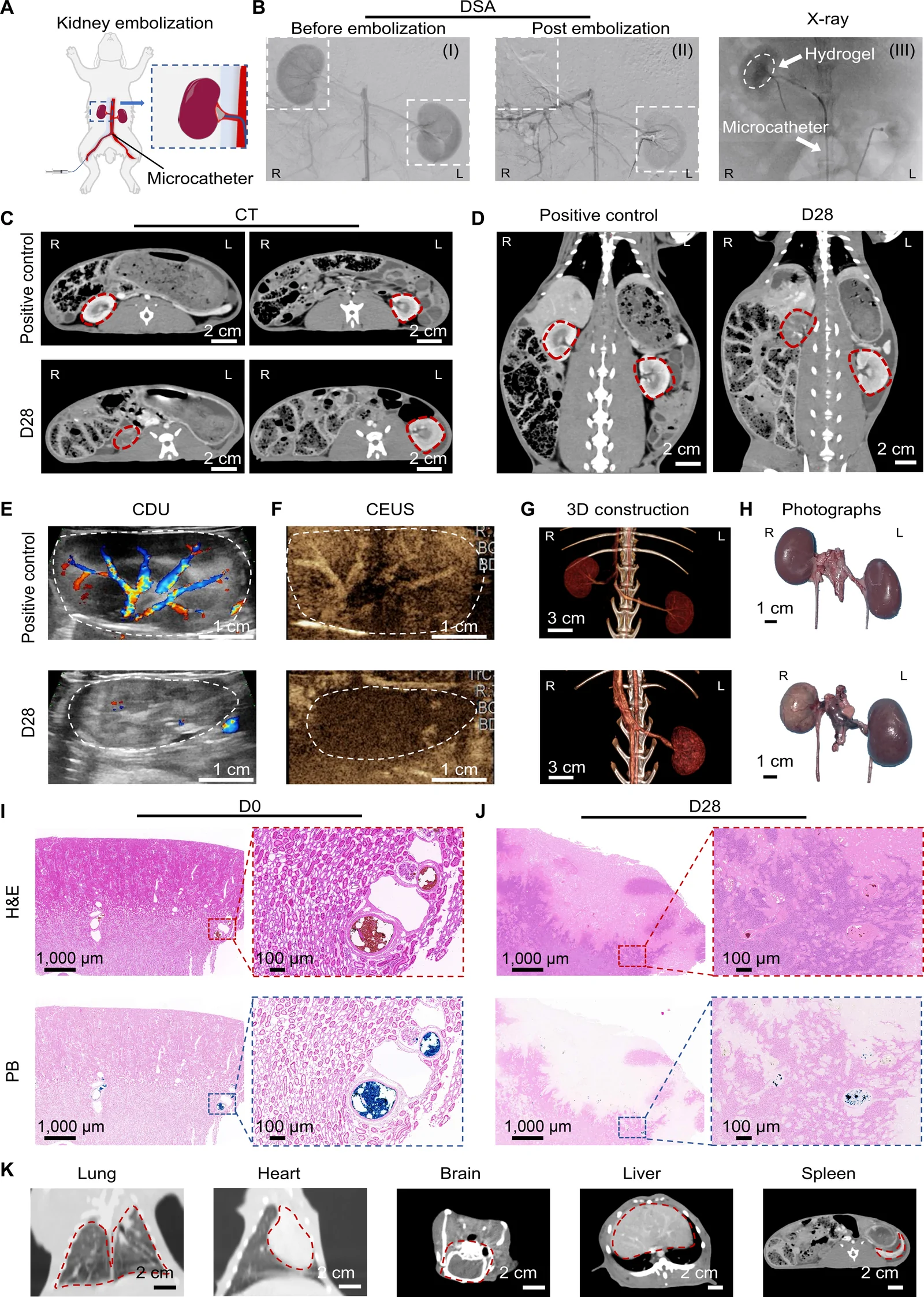
Assessment of the embolization effect of C_1.0_AMG in rabbit kidney. (A) Illustration of renal embolization in a rabbit model (*n* = 5). (B) DSA images of the renal arteries (I) before and (II) post-embolization and (III) x-ray image of injecting C_1.0_AMG-I. (C) Transverse CT, (D) coronal CT, (E) CDU, (F) CEUS images, (G) 3-dimensional (3D) construction, and (H) photographs of rabbit kidneys in the positive control group and D28 post-embolization. H&E staining images and PB staining images of rabbit kidneys in the (I) D0 and (J) D28 post-embolization. (K) CT images of lung, heart, brain, liver, and spleen in experimental rabbit.

The vascular supply and blood perfusion of the embolized kidney were then assessed using color Doppler ultrasound (CDU) and contrast-enhanced ultrasound (CEUS). The CDU images revealed the absence of blood flow signals in the arteries and its branches in the embolized kidney (Fig. 5E), and the lack of blood perfusion in the atrophied kidney was also confirmed by CEUS (Fig. 5F). Moreover, the 3D construction demonstrated that the right kidney remained invisible after embolization, confirming a long-lasting embolization effect (Fig. 5G). Following these analyses, the rabbits were dissected and the volume of the embolized right kidney was evaluated, confirming that this had shrunk significantly (Fig. 5H). In addition, residual hydrogel was observed in the transverse section of the anatomical image of the embolized kidney vessels, confirming the retained presence of hydrogel (Fig. S18). Furthermore, the renal parenchyma was assessed before and after embolization using hematoxylin and eosin (H&E) and Prussian blue (PB) staining. The results indicated that on the 28th day, the nuclei in the kidney tissue showed significant deformation, atrophy, and even disappearance, along with extensive necrosis, compared with the observations recorded on day 0 (Fig. 5I). Furthermore, despite the decrease in mass of subcutaneously implanted CAMG in mice from ~100 mg to 57.8 ± 3.6 mg and the occurrence of internal structural collapse (Fig. S19A and B), the embolic efficacy was still retained. On days 0 and 28, the blood vessels were found to be clearly filled with hydrogel (Fig. 5J), demonstrating the excellent vascular deliverability and the long-term embolization effect of the hydrogel in rabbit renal artery embolization. A whole-body CT scan confirmed that the other vital organs remained normal, including the lungs, liver, spleen, heart, and brain (Fig. 5K), indicating the absence of ectopic embolization. As shown in Fig. S19C, serum alanine aminotransferase (ALT) and aspartate aminotransferase (AST) levels typically increased after embolization, whereas albumin (ALB) and total bilirubin (TBIL) levels remained stable. H&E staining of the major organs revealed no notable morphological abnormalities in any treatment group (Fig. S19D). Collectively, these results demonstrate the biocompatibility of the CAMG hydrogel.

### In vivo arterial embolization and magnetic hyperthermia in rabbit with orthotopic HCC

Magnetic hyperthermia (MHT) is an emerging cancer treatment technology that utilizes magnetic materials to generate heat within tumors under an AMF, resulting in effective therapeutic outcomes [56]. As a noninvasive method with low toxicity, MHT is not constrained by tissue penetration limitations, making it applicable to various cancer types [57,58]. Incorporating MHT into TAE regimens has the potential to significantly improve the therapeutic effectiveness of TAE. To evaluate the magnetic heating effects of C_1.0_AMG, the hydrogel was exposed to an AMF. Under magnetic field conditions of 15 and 25 kA m^−1^, the hydrogel temperature rapidly increased to 60 °C within 2 min (Fig. S20A and B), producing a thermal effect that can potentially kill cancer cells. Notably, the temperature increase cycle shown in Fig. S20C clearly illustrates the magnetic heating stability of C_1.0_AMG. These results confirm the excellent magnetic heating effect of C_1.0_AMG and possess the potential to kill tumor cells.

To assess the in vivo efficacy of C_1.0_AMG in treating HCC, a rabbit model with orthotopic HCC was used. Under real-time x-ray fluoroscopy guidance provided by a DSA machine, the microcatheter was inserted into the hepatic artery. Subsequently, iodixanol was injected into the blood vessels to confirm the location and contour of the tumor (green circles and arrows) as shown in DSA and x-ray image (Fig. 6B). Once successful catheterization was achieved, the freshly prepared CAMG hydrogel was smoothly delivered through a 2.6-F microcatheter, and the entire delivery-to-embolization process could be completed within a 4-min time window, even for tortuous, distal tumor-feeding vessels. Post-embolization, iodixanol was injected to confirm occlusion of the tumor-feeding artery. An angiogram showed no perfusion to the tumor’s blood supply artery, indicating successful vessel occlusion by the hydrogel (Fig. 6C). The rabbits in the CAMG + MHT group were treated 3 times with MHT under an AMF after hydrogel injection. CT imaging and 3D reconstruction revealed reductions in tumor volume in both the Lipiodol + doxorubicin hydrochloride (DOX) and CAMG + MHT treatment groups. The Lipiodol + DOX group showed a reduction of 30.2 ± 7.3%, whereas the CAMG + MHT group demonstrated a significantly enhanced reduction of 62.4 ± 6.3%. These results demonstrate that in transarterial interventional embolization, the CAMG + MHT therapy exhibits superior efficacy in inhibiting tumor growth compared to the conventional lipiodol + DOX regimen. CT images show that after MHT treatment, the peritumoral liver parenchyma exhibits no abnormal imaging signs such as necrosis or obvious inflammation (which typically manifest as well-defined hypodense areas without enhancement on contrast-enhanced scans), indicating that this therapy does not cause significant damage to surrounding tissues and holds promise as a safe new strategy for tumor thermal ablation. Histological staining was then employed to evaluate the detailed conditions of the hepatic tissue cells and the distribution of the hydrogel within the blood vessels. As shown in Fig. S21 and Fig. 6G, H&E staining of the hepatic tissue revealed a large number of live tumor cells in the untreated group. Furthermore, the majority of tumor cell nuclei were deformed and apoptotic, and extensive tissue necrosis was evident in the CAMG + MHT group compared to the TAE group, which was attributed to the lack of blood perfusion to the tumor site and the effect of MHT. PB staining images showed that the CAMG + MHT group retained a significant amount of hydrogel inside the differently sized tumor blood vessels (blue and red arrows), indicating its long-lasting embolization effect in the tumor vascular (Fig. 6I). Overall, the designed bioinspired reinforced CAMG exhibited catheter injectability and achieved intravascular solidification, thereby facilitating long-term embolization.

**Fig. 6.**
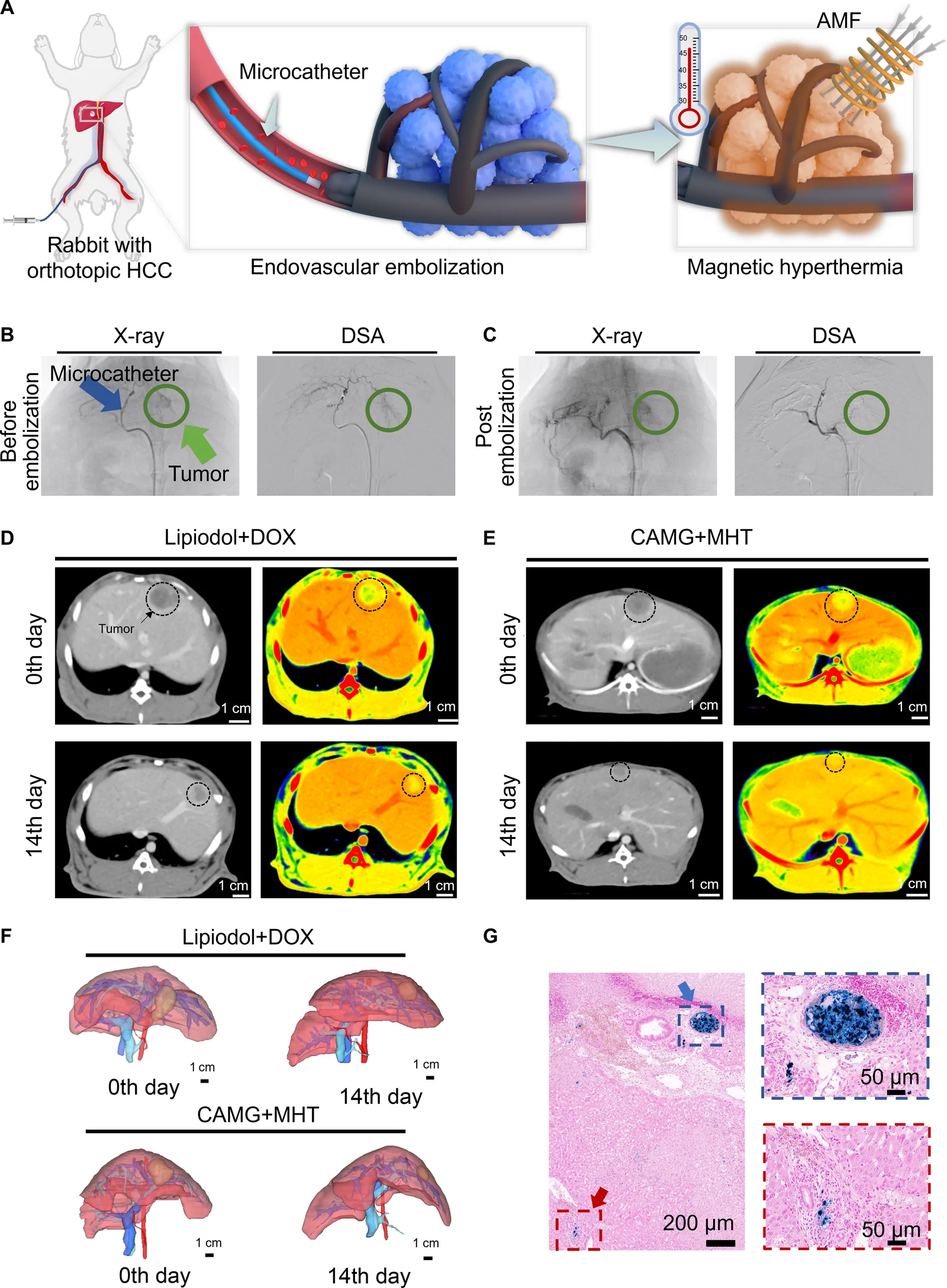
Magnetically induced heating performance of hydrogel and in vivo therapeutic effect evaluation in rabbit with orthotopic HCC. (A) Schematic diagram of TAE and MHT in the rabbit with orthotopic HCC (*n* = 5). X-ray and DSA images of the rabbits (B) before and (C) post-embolization. Blue arrow indicated the location of the microcatheter. Green circle and arrow displayed the tumor location. (D to F) The representative CT images and (D) corresponding 3D reconstructions were obtained for rabbits subjected to various treatments. The yellow area represents a tumor. (G) PB staining of residual lesions for the CAMG + MHT group. The blue and red arrows indicated the location of the hydrogel in blood vessels of different sizes.

## Conclusion

In conclusion, inspired by the process that wheat roots secrete organic acids to degrade calcium carbonate in the soil, absorb calcium ions, and strengthen the cell wall, a bioinspired reinforced hydrogel was developed, demonstrating enhanced mechanical properties, making it suitable for embolizing tumor blood vessels in combination with MHT. The reinforced MCG was formed using a bottom-up self-assembly strategy. In this system, the gaps between the spherical nanoparticles aggregates were filled with the natural polysaccharide Alg. Upon chelation with Ca^2+^, abundant cross-links formed, thereby enhancing the mechanical properties of the original colloidal gel system. The slow hydrolysis of the GDL produced H^+^, which acidified the CaCO_3_ NPs to produce Ca^2+^, chelating with Alg and exhibiting a process like hardening of wheat cell wall. In vitro and in vivo experiments demonstrated that this strategy enhanced the mechanical properties of electrostatically assembled colloidal gels, leading to excellent embolic effects, which were further amplified upon combination with MHT. Overall, this approach provides new opportunities and strategies for the future clinical treatment of HCC.

## Materials and Methods

### Materials

Gelatin A (from porcine skin, 300 Bloom) and poly (acrylic acid) (PAA; *M*_w_ ~ 1,800) were purchased from Sigma-Aldrich Co. Ltd. Acetone (≥99.0%), iron (III) chloride hexahydrate (FeCl_3_·6H_2_O; ≥99.9%), glycine (≥99.0%), ethylene glycol (EG; ≥99.0%), diethylene glycol (DEG; ≥99.0%), sodium acetate (NaAc; ≥99.0%), calcium chloride dihydrate (CaCl_2_·2H_2_O; ≥99.0%), ethanol (≥99.7%), hydrochloric acid (AR; 36.0% to 38.0%), ammonium hydrogen carbonate (NH_4_HCO_3_; ≥99.0%), and Alg were obtained from Sinopharm Chemical Reagent Co. Ltd. GDL (≥99.0%) was purchased from Aladdin Reagent Co. Ltd. Glutaraldehyde (GA; 25 wt % solution in water) was commercially available from Acros Organics.

### Characterizations

SEM images were obtained using a scanning electron microscope (Zeiss Supra 40, Germany). The particle size was evaluated by a dynamic light scattering instrument (Malvern Zetasizer, Nano-ZS90, UK). XRD pattern was obtained using a Philips X’ Pert Pro SUPER instrument (Netherlands). Rheological characterizations were performed by a rotational rheometer (Discovery HR-30, US). The injection force of hydrogel was measured using a mechanical testing system (Instron 5965, USA). The MHT was performed using high-frequency (312 kHz) heating equipment (Shuangping SPG, China).

### Synthesis of GNPs

GNPs were synthesized by a 2-step desolvation method using water and acetone (*1*). In the initial step, 50 g of gelatin A powder was dissolved in 1 l of distilled water at 50 °C. Then, 1 l of acetone was added to the gelatin solution, and the mixture was left at room temperature for 1 h to precipitate high-molecular-weight gelatin. After that, the supernatant was discarded, and the gelatin precipitates were dissolved in distilled water and freeze-dried for later use. In the second step, 3.75 g of the freeze-dried gelatin was dissolved in 75 ml of distilled water, and the pH of the solution was adjusted to 2.3 by adding hydrochloric acid. Next, 225 ml of acetone was added to the gelatin solution at 40 °C with a syringe pump at a dripping rate of 4 ml/min while vigorously stirring the solution at 1,200 rpm. To stabilize the GNPs by cross-linking, 550 μl of 25 wt % GA solution was added to the gelatin dispersion at room temperature and left to react overnight with stirring at 600 rpm. The next day, 300 ml of a 100 mM aqueous solution of guanidine hydrochloride was added to the gelatin dispersion to block unreacted aldehyde groups and make the GNPs positively charged. After 2 h of stirring, the dispersion was filtered through a nylon mesh (100-μm mesh size). Finally, it was centrifuged and dispersed in water for standby use.

### Synthesis of PAA-coated ION nanoparticle

The PAA-coated ION nanoparticles were synthesized via a one-pot solvothermal reaction (*2*). For instance, under magnetic stirring, 4 mmol of FeCl_3_·6H_2_O, 0.1 g of PAA, and 6 g of NaAc were dissolved in a mixed solution of 10 ml of EG and 30 ml of DEG. The obtained homogeneous yellow solution was transferred to a stainless-steel autoclave lined with Teflon, sealed, and then heated at 200 °C. After reacting for 12 h, the autoclave was cooled to room temperature. The obtained IONs were washed repeatedly with ethanol and deionized water. Finally, they were dispersed in deionized water for standby use.

### Synthesis of CaCO_3_ nanoparticles

CaCO_3_ nanoparticles (CaCO_3_ NPs) were synthesized by a gas diffusion process (*3*). Briefly, 150 mg of CaCl_2_·2H_2_O was dissolved in a beaker containing 100 ml of anhydrous ethanol; the mixture was then placed in a sealed container with 5 g of NH_4_HCO_3_ and kept at 40 °C. After 24 h, the white CaCO_3_ NPs were collected and purified by centrifuging at 8,000 rpm. Finally, the obtained CaCO_3_ NPs were dispersed in ethanol and freeze-dried for further use.

### Preparation of MCG, AMG, and C*_x_*AMG

The preparation of MCG was based on the previous work (*4*,*5*). The suspensions of GNPs and IONs were mixed using a vortex mixer, and then GDL was added. Typically, the abovementioned nanoparticles were dispersed in an alkaline aqueous solution (pH ≈ 11) to obtain homogeneous suspensions. The obtained ION suspension was added to the GNP suspension and mixed in a plastic tube by vortex oscillation, and the final concentrations were 2% and 8% w/v, respectively. Subsequently, the GDL powder (80 mM) was dissolved in the abovementioned mixture. The preparation process of AMG was similar to that of MCG. The concentration of Alg in the final system is 2% w/v. C*_x_*AMG (*x* = 0.5, 1.0, and 1.5) was prepared by adding CaCO_3_ NPs in AMG, and the content of CaCO_3_ NPs was 0.5%, 1.0%, and 1.5% w/v, respectively. Additional GDL was added, and the molar ratio of GDL to CaCO_3_ NPs is 2:1.

### Rheological characterizations

The rheological properties of hydrogel were tested by a rotational rheometer with a rotating plate and a gap distance of 1,000 μm. Frequency sweep measurements were performed at frequencies ranging from 0.1 to 10 Hz at a fixed strain of 1%. Viscosity test measurement (shear rate from 0.1 s^−1^ to 10 s^−1^) at the fixed strain. Peak-hold test was carried out with alternating strain between different shear rates (s^−1^) in sequential order: 0.01 (60 s), 1,140 (5 s), and 0.01 (115 s) to monitor the shear recovery of the gels. During the time sweep method, the gelation behavior of MCG and CAMG was tested for 2 h. Strain sweep measurements were performed at a constant frequency of 1 Hz with a strain range of 1% to 100%. Self-healing properties of the gels were performed under different oscillation strains for 5 cycles. All hydrogels to be tested were extruded by syringe and transferred to the rheometer stage. All the tests were under the 25 °C environment.

### Injection performance of CAMG

CAMG was loaded into a 1-ml plastic syringe and tested for its injection force through different types of microcatheters (2.6 and 4 F). The plunger was pressed down using a pressure plate, and the force on the plunger was recorded by the weighing sensor. All tests were conducted on a Material Test System (Instron, 5965).

### In vitro embolization pressure test

The pipeline was assembled according to the schematic diagram in Fig. 3A (inner diameter = 2 mm). The 200-μl hydrogel was injected into the tube before using a syringe pump to inject the anticoagulant blood into the test model (flow rate = 50 ml min^−1^). When the embolic agent is flushed out, we record the pressure as the embolization pressure.

### In vitro embolization in simulated blood circulation system

The in vitro simulated blood circulation system comprised a mannequin integrated with blood pump, fluid reservoir, blood pressure monitor, 3D vascular systems (including common hepatic artery, right and left hepatic arteries, FA, and inferior vena cava), and liver tumor. Agarose ball was used as tumor models and filled with silicone tubes with an inner diameter of 300 μm to simulate complex vascular channels. When the fluid pressure in the pipeline reached 16 kPa, 200 μl of C_1.0_AMG was injected into the simulated tumor blood vessel through a 2.6-F catheter. After a certain period, we assessed the patency of the simulated blood vessel to evaluate the embolic efficacy of the hydrogel.

### MHT effect of C_1.0_AMG

The magnetic heating performance was evaluated using a high-frequency heating machine (Shuangping SPG, China). Hydrogel samples were placed within the coil and tested for their heating effect under different AMFs (*f* = 312 kHz). Thermal images of the samples were captured at specific time intervals using an infrared thermal imaging camera (Fluke Ti400, USA).

### In vivo renal artery embolization in a rabbit model

All experimental rabbits (*n* = 5) were anesthetized with isoflurane. Initially, the right FA was bluntly dissected and suspended. Subsequently, an incision was made on the FA, and an arterial sheath was inserted. A 4-F microcatheter (451-443H0, Cordis, USA) was then advanced into the abdominal aorta through the sheath. Under real-time x-ray fluoroscopy guidance provided by a DSA machine (Artis zee III ceiling, SIEMENS, Germany), the microcatheter was further inserted into the left renal artery. Following this, iodixanol (0.4 ml) was deployed into blood vessels to confirm the location of the catheter tip and the patency of the renal artery. Subsequently, 0.3 ml of C_1.0_AMG was slowly deployed through the microcatheter to embolize the renal artery, with real-time x-ray monitoring. On the 28th day, CT scanning was used to detect changes in the organs. Additionally, CDU and CEUS (ARIETTA 70, Hitachi, Japan) were employed to detect the blood signal of the embolized renal arteries. Furthermore, the embolized kidney was fixed in formalin for 24 h, dehydrated, and then embedded in paraffin.

### In vivo arterial embolization and magnetic heating treatment in rabbit with orthotopic HCC

All experimental rabbits (*n* = 5) were anesthetized with isoflurane. Initially, the right FA was bluntly dissected and suspended. Subsequently, an incision was made on the FA, and an arterial sheath was inserted. A 2.6-F microcatheter (STD125-26S, ASAHI, Japan) was then advanced into the abdominal aorta through the sheath. Under real-time x-ray fluoroscopy guidance provided by a DSA machine (Artis zee III ceiling, SIEMENS, Germany), the microcatheter was further inserted into the hepatic artery supplying the liver tumor. Following this, iodixanol (0.4 ml) was deployed into blood vessels to confirm the location of the catheter tip. Subsequently, 0.2 ml of C_1.0_AMG was slowly injected through the microcatheter to embolize the artery with real-time x-ray monitoring. On the 2nd, 6th, and 12th days, the rabbits scheduled for TAE treatment will be subjected to MHT under an AMF. Meanwhile, to establish a more clinically relevant efficacy benchmark, this study compared the novel CAMG + MHT therapy with conventional lipiodol + DOX therapy in a rabbit model of HCC. On day 14, the rabbits were euthanized to harvest liver tumor tissues. The embolized liver lobes were fixed in 10% formalin for 24 h, dehydrated, and embedded in paraffin.

### Statistical analysis

All the experiments were repeated for at least 3 parallel samples, and experimental data were presented as mean ± SD. The SPSS software with a version of 24.0 was employed for the statistical analysis with analysis of variance (ANOVA) for multiple-group comparisons (*P ≤ 0.05, **P ≤ 0.01, and ***P ≤ 0.001).

## Ethical Approval

All animal experiments were conducted according to the recommendations of the National Institution of Health Guidelines for the Care and Use of Laboratory Animals, which were approved by the Institutional Animal Care and Use Committee of Hefei University of Technology (no. HFUT20201101001).

## Data Availability

The data supporting this study’s findings are available from the corresponding author upon reasonable request.
