# Supplementary material for: Bioinspired Reinforcement of Magnetic Colloidal Gel for the Embolization of Hepatocellular Carcinoma
Source: Research (Wash D C). 2026 Oct 23;9:1446. doi: 10.34133/research.1446 (PMC13691249; doi:10.34133/research.1446)
Supplement: Supplementary file 1 [file research.1446.f1.docx]

Supplementary Materials for

**Bioinspired Reinforcement of Magnetic Colloidal Gel for the Embolization of Hepatocellular Carcinoma**

Chaowei Lu^1^†, Guangyi Luo^2^†, Huai Yang^1^†, Jinlong Hu^2^, Xu Yan^1^*, Liang Dong^3^, Baoqiang Cao^2^*, Yang Lu^1^*

Corresponding author. Xu Yan, yanxu@hfut.edu.cn; Baoqiang Cao: caobaoqiang@126.com; Yang Lu, yanglu@hfut.edu.cn

**The PDF file includes:**

Figs. S1 to S21

Reference

**Fig. S1.** **Characterization of GNPs.** A) SEM image and B) hydrated particle size of GNPs.

**Fig. S2. Characterization of IONs.** A) SEM image, B) TEM image and C) hydrated particle size of IONs.

**Fig. S3. Characterization of CaCO_3_ NPs.** A) SEM image, B) TEM image and C) particle size of CaCO_3_ NPs.

**Fig. S4. Preparation of CAMG.**

**Fig. S5. Element mapping analysis of CAMG.** SEM image of the C_1.0_AMG and the corresponding elemental mapping analysis A) at 0 min and B) 120 min. Scale bar: 5 μm.

**Fig. S6. The magnetic response of CAMG.** CAMG was injected into PBS solution without or with magnet.

**Fig. S7. Injection force test of AMG through 2.6 F catheter.** Injection force test of different Alg content (1, 2 and 3 w/v%) in AMG.

**Fig. S8.** **Embolic pressure test of AMG.** Embolic pressure of different Alg content (1, 2 and 3 w/v%) in AMG at 0 min.

**Fig. S9. Shear-thinning test of hydrogel.** The viscosity of MCG, AMG, C_0.5_AMG, C_1.0_AMG and C_1.5_AMG versus shear rate at 0 min.

**Fig. S10. Diagram of injection force testing device.**

**Fig. S11. Injection force test through catheter of CAMG.** Injection force of C_0.5_AMG, C_1.0_AMG, C_1.5_AMG through C) 4F catheter and D) 2.6F catheter at 0 min.

**Fig. S12. Modulus test at different frequencies of hydrogel.** G' and G" of MCG, AMG, C_0.5_AMG, C_1.0_AMG, C_1.5_AMG at A) 0 min and B) 120 min under frequency sweep.

**Fig. S13.** **Modulus test at different strain of CAMG.** G′ and G" on strain amplitude sweep for C_0.5_AMG, C_1.0_AMG, C_1.5_AMG at 120 min.

**Fig. S14. SEM of C_1.0_AMG.** The internal structure of the C_1.0_AMG after freeze-drying at different time points.

**Fig. S15. pH change versus time.** The pH changes with time in GDL and CaCO_3_ NPs mixed solution.

**Fig. S16. XRD of hydrogel.** A) XRD analysis of IONs and CaCO_3_ NPs. XRD analysis of C_0.5_AMG, C_1.0_AMG, C_1.5_AMG at B) 0 min and C) 120 min.

**Fig. S17. Characterization of C_1.0_AMG-I.** A) X-ray images of different samples. B) G' and G" of C_1.0_AMG and C_1.0_AMG-I under frequency sweep. C) Modulus over time of C_1.0_AMG-I. D) Injection force test of C_1.0_AMG-I at 0 min. (C_1.0_AMG-I represented the gel matrix contained 10 w/v% iodixanol)

**Fig. S18. Imagery data of embolized rabbit.** Cross section images of kidney with normal or embolized kidney. White arrows present the location of CAMG.

**Fig. S19. *In vivo* degradation** **and biosafety experiments of hydrogels.** A-B) In vivo degradation rate of CAMG hydrogel (n=5). C) Comparison of major liver function indices in tumor-bearing rabbits before and after treatment (n=5). D) Hematoxylin and eosin (H&E) staining was performed on major organs from these rabbit s (n=5).

**Fig. S20. Magnetic hyperthermia performance *in vitro*.** A) Temperature changes of C_1.0_AMG as a function of time under different AMF (*H* = 15 and 25 kA m^−1^) and B) corresponding infrared thermal images. C) Analysis of the stability of magnetic hyperthermia performance with intermittent AMF treatment (*H* = 15 kA m^−1^, 3 cycles).

**Fig. S21. Magnetic hyperthermia performance *in vivo*.** H&E staining of residual lesions for the untreated, TAE and TAE+MHT groups.

**Reference**

1. M. Diba, H. Wang, T. E. Kodger, S. Parsa, S. C. G. Leeuwenburgh, Highly Elastic and Self‐Healing Composite Colloidal Gels. *Adv. Mater.* **29**, 1604672 (2017).
2. J. Zhang, Q. Fang, J. Duan, H. Xu, H. Xu, S. Xuan, Magnetically Separable Nanocatalyst with the Fe_3_O_4_ Core and Polydopamine-Sandwiched Au Nanocrystal Shell. *Langmuir* **34**, 4298–4306 (2018).
3. Z. Dong, L. Feng, Y. Hao, M. Chen, M. Gao, Y. Chao, H. Zhao, W. Zhu, J. Liu, C. Liang, Q. Zhang, Z. Liu, Synthesis of Hollow Biomineralized CaCO_3_–Polydopamine Nanoparticles for Multimodal Imaging-Guided Cancer Photodynamic Therapy with Reduced Skin Photosensitivity. *J. Am. Chem. Soc.* **140**, 2165–2178 (2018).
4. Y. Du, X. Yan, W. Wu, B. Cao, J. Hu, J. Xue, Y. Lu, Magnetic and pH dual responsive injectable magnetic zeolitic imidazole framework based colloidal gel for interventional treatment on hepatocellular carcinoma. *Chem. Eng. J.* **497**, 154180 (2024).
5. W.-S. Wu, X. Yan, S. Chen, Y.-X. Du, J.-L. Hu, Y. Song, Z.-B. Zha, Y.-J. Xu, B.-Q. Cao, S.-H. Xuan, X.-Y. Liu, B. Chen, L. Dong, Y. Lu, S.-H. Yu, Minimally Invasive Delivery of Percutaneous Ablation Agent via Magnetic Colloidal Hydrogel Injection for Treatment of Hepatocellular Carcinoma. *Adv. Mater.* **36**, 2309770 (2024).
